# Overcoming Understaging and Undergrading in Upper Tract Urothelial Carcinoma. Comment on Ghoreifi et al. Modern Kidney-Sparing Management of Upper Tract Urothelial Carcinoma. *Cancers* 2023, *15*, 4495

**DOI:** 10.3390/cancers16051002

**Published:** 2024-02-29

**Authors:** Joshua S. Jue, Mahmoud Alameddine, Noel A. Armenakas

**Affiliations:** 1 Department of Urology, Lenox Hill Hospital, Northwell Health, Zucker School of Medicine at Hofstra/Northwell, New York, NY 10075, USA; drarmenakas@gmail.com; 2 Department of Urology, Ottumwa Regional Health Center, Ottumwa, IA 52501, USA; dralameddine@hotmail.com

Kidney-sparing management for upper tract urothelial carcinoma (UTUC) has become more common but is still most limited by inaccurate histopathologic diagnosis. Ureteroscopy is the preferred method of pathologic diagnosis and staging but is limited by high rates of disease undergrading and understaging. In a meta-analysis, staging could not be performed in 32% of patients, and the cumulative nondiagnostic ureteroscopic biopsy rate was 8%, with some individual series rates as high as 64% [1]. This inability to achieve a pathologic diagnosis or stage can delay nephron-sparing therapies for low-grade and low-stage disease and contribute to disease progression. Substantial cumulative understaging in 46% and undergrading in 29% of patients still occurred when biopsies were successfully performed. Endoscopic ablation and segmental ureterectomy were discussed as the primary kidney-sparing surgical techniques in a recent review article discussing kidney-sparing management of UTUC [2]. Although both remain surgical options, segmental ureterectomy is a substantially more morbid and invasive operation compared to endoscopic ablation. Conversely, endoscopic ablation is often an outpatient procedure but is severely limited by the aforementioned understaging and undergrading.

High-grade disease and subepithelial connective tissue invasion (T1 disease) are important to diagnose on the initial biopsy because they guide UTUC management and yield important prognostic information. Muscle invasion (T2 disease) on a nephroureterectomy specimen is highly predicted by the combination of high-grade disease and ≥T1 stage at the time of biopsy [3]. Although the phase III POUT trial showed improved disease-free survival with adjuvant chemotherapy for locally advanced UTUC [4], only 15% of nephroureterectomy patients remain eligible for cisplatin-based chemotherapy after surgery [5]. However, a recent phase II trial demonstrated a pathologic response with improved survival outcomes after neoadjuvant gemcitabine and split-dose cisplatin [6]. Progression-free and overall survival rates at 5 years were 72% and 79%, respectively [6]. Patients with partial and complete pathologic response were associated with significantly improved progression-free and overall survival compared to non-responders [6]. This level 1 evidence underscores the importance of identifying high-risk UTUC prior to nephroureterectomy.

Advancements in preoperative imaging to improve UTUC clinical staging can be further enhanced by endoscopic surgical techniques that are not solely ablative. Bladder tumors and prostate adenomas are increasingly resected endoscopically by en bloc enucleation, making this technique a natural extension for UTUC. The complete resection of large upper tract tumors with extensive subepithelial connective tissue along the tumor base can be safely performed through ureteroscopic en bloc enucleation [7]. This technique is performed by entering the subepithelial connective tissue plane with low ablation laser settings and maintaining this plane through laminar irrigation from the ureteroscope [7]. Although this is a new technique, upper tract tumor en bloc enucleation overcomes the limitations of current endoscopic biopsy and ablative techniques to improve diagnostic and therapeutic potential. This endoscopic technique underscores the ability to perform a thorough upper tract tumor resection using a basic ureteroscope and laser available to most urologists in an ambulatory setting.
